# Necrosis and apoptotic index as prognostic factors in non-small cell lung carcinoma: a review

**DOI:** 10.1186/2193-1801-3-120

**Published:** 2014-03-01

**Authors:** Charalampos Gkogkou, Konstantina Frangia, Muhammad W Saif, Rodoula Trigidou, Konstantinos Syrigos

**Affiliations:** Pathology Department, “SOTIRIA” General Hospital, Athens, Greece; Division of Hematology/Oncology, Tufts Medical Center, Boston, USA; Oncology Unit GPP, “SOTIRIA” General Hospital, Athens School of Medicine, Athens, Greece; Yale School of Medicine, New Haven, USA

**Keywords:** Apoptotic index, Necrosis, Non-small cell lung carcinoma (NSCLC), Prognosis

## Abstract

Necrosis and apoptosis represent two pathogenetically distinct types of cell death. Necrosis is associated with pathologic conditions while apoptosis is a physiological process of programmed cell death, which is associated with normal tissue growth and is frequently impaired in various forms of cancer. Tumor necrosis and apoptotic index (AI) have been previously evaluated as prognostic biomarkers in lung cancer, but their exact clinical value remains unclear. The aim of this study was to perform a systematic review of the MEDLINE literature on the prognostic significance of these histopathological markers in patients with non-small cell lung carcinoma (NSCLC). Although a substantial body of evidence suggests that tumor necrosis may be a strong predictor of aggressive tumor behavior and reduced survival in patients with NSCLC, the independent prognostic value of this biomarker remains to be firmly established. Furthermore, previous data on the prognostic significance of apoptotic index in NSCLC are relatively limited and largely controversial. More prospective studies are necessary in order to further validate tumor necrosis and AI as prognostic markers in NSCLC.

## Introduction

Lung cancer remains the leading cause of death from cancer worldwide and one of the 10 leading causes of death from all causes (Murray & Lopez 2013). The prognosis of lung cancer remains poor. Even in the cases of resected tumors, recurrence rates are high. The identification of independent and reproducible prognostic factors may affect therapeutic decisions and influence clinical research. Histopathologic features are commonly evaluated for their potential use as prognostic factors. Tumor necrosis and the apoptotic index (i.e. the proportion of apoptotic cells in tumor specimens) are two such features.

Cells maintain homeostasis by regulating a wide range of both intracellular and extracellular microenvironmental parameters. Various stress factors can force cells to adapt through hypertrophy, hyperplasia, atrophy or metaplasia (Kumar et al. 2007), achieving stability and -sometimes- normal function. If the external stress factor is persistent or severely harmful, the cells’ adjustment mechanisms are not sufficient to prevent cell injury. In that case, cell changes may be partially reversible, and there is always the possibility for a cell to overcome the stressful condition and survive without residual malfunction. On the other hand, a long-standing harmful stressor can lead to irreversible damage and cell death.

There are two distinct types of cell death in terms of pathogenesis: necrosis and apoptosis. Necrosis is always associated with abnormal processes, such as exposure to toxins, various infections, trauma and ischemia. Cell injury leads to membrane dysfunction, leak of lysosomic enzymes in the cytoplasm and, finally, cell digestion (Poon et al. 2010). Secondary inflammation occurs in the surrounding tissue, and neutrophilic enzymes further contribute to the evolving necrosis (Proskuryakov & Gabai 2010). Depending on the causative agent, several different types of necrosis have been described. Specifically, in case of a tumor mass, the pattern of coagulative necrosis is the most common. It is mainly caused by tissue hypoxia due to rapid tumor growth, insufficient neovascularization, compression and thrombotic obstruction of adjacent vessels. Resulting necrosis is characterized by the presence of dead cells in the form of anucleate “ghost cells’, but with preservation of the tissue architecture (Kumar et al. 2007). Coagulative necrosis is often massive and grossly evident in malignant neoplasms, although necrotic foci are also described in a minority of benign tumors.

In contrast to necrosis, apoptosis is a physiological phenomenon that occurs spontaneously and is associated with normal tissue growth. It is an active process, linked to specific molecular pathways (Edinger & Thompson 2004). It discards independent, severely injured cells without causing an inflammatory host reaction. Factors such as hypoxia, irradiation or chemotherapeutic agents can cause irreversible DNA damage and activate the apoptotic process, thus diminishing the risk of a possibly harmful mutation and malignant transformation (Kumar et al. 2007). A characteristic feature of cancer cells is their ability to escape apoptosis.

The prognostic significance of tumor necrosis and apoptotic index (AI) in NSCLC has been previously investigated in several clinicopathological studies spanning the past three decades. Nevertheless, most of these previous reports have yielded variable or even controversial results. The aim of this study was to perform a review of the MEDLINE literature in an attempt to determine the exact clinical value of tumor necrosis and apoptotic index (AI) as prognostic biomarkers in NSCLC. We retrieved 28 papers, investigating the prognostic significance of necrosis and/or apoptosis in patients with primary NSCLC (Tables 1 and 2) (Lipford et al. 1984; Elson et al. 1988; Lee et al. 1989; Roeslin et al. 1991; Roberts et al. 1992; Shahab et al. 1992; Tormanen et al. 1995; Kessler et al. 1996; Goldstein et al. 1999; Swinson et al. 2002; Poleri et al. 2003; Khan et al. 2004; Rena et al. 2007; Inoue et al. 2008; Cho et al. 2008; Sardari Nia et al. 2010; Kiliçgün et al. 2010; Park et al. 2011; Pataer et al. 2012; Tantraworasin et al. 2013; Komaki et al. 1996; Tanaka et al. 1999; Matturi et al. 1999; Langendijk et al. 2000; Gorgoulis et al. 2000; Hwang et al. 2001; Puglisi et al. 2002; Dworakowska et al. 2009). Studies on apoptosis-related genes were excluded.

**Table 1:** **Studies on the prognostic role of necrosis in patients with non-small cell lung carcinoma (listed in chronological order)**

***Ref no***	***Type***	***Year***	***No of patients***
Lipford et al. (1984)	*retrospective*	1984	173
Elson et al. (1988)	*retrospective*	1988	47
Lee et al. (1989)	*retrospective*	1989	30
Roeslin et al. (1991)	*retrospective*	1991	97
Roberts et al. (1992)	*retrospective*	1992	87
Shahab et al. (1992)	*retrospective*	1992	28
Tormanen et al. (1995)	*retrospective*	1995	75
Kessler et al. (1996)	*retrospective*	1996	593
Goldstein et al. (1999)	*retrospective*	1999	218
Swinson et al. (2002)	*retrospective*	2002	178
Poleri et al. (2003)	*retrospective*	2003	53
Khan et al. (2004)	*retrospective*	2004	98
Rena et al. (2007)	*retrospective*	2007	87
Inoue et al. (2008)	*retrospective*	2008	97
Cho et al. (2008)	*prospective*	2008	55
Nia et al. (2010)	*retrospective*	2010	239
Kilinçgün et al. (2010)	*N/A*	2010	152
Park et al. (2011)	*retrospective*	2011	201
Pataer et al. (2012)	*retrospective*	2012	358
Tantraworasin et al. (2013)	*retrospective*	2013	227

*N/A: Not Available.*

**Table 2:** **Studies on the prognostic role of apoptosis in patients with non-small cell lung carcinoma (listed in chronological order)**

***Ref no***	***Type***	***Year***	***No of patients***
Tormanen et al. (1995)	*retrospective*	1995	75
Komaki et al. (1996)	*retrospective*	1996	173
Tanaka et al. (1999)	*retrospective*	1999	236
Matturi et al. (1999)	*N/A*	1999	38
Langendijk et al. (2000)	*retrospective*	2000	161
Gorgoulis et al. (2000)	*retrospective*	2000	63
Hwang et al. (2001)	*retrospective*	2001	68
Puglisi et al. (2002)	*retrospective*	2002	N/A
Dworakowska et al. (2009)	*retrospective*	2009	170

*N/A: Not Available.*

## Review

### Tumor necrosis

Tumor necrosis is a histopathologic feature commonly found in resected pulmonary tumors. The identification of tumor necrosis as an independent and significant prognostic factor could affect therapeutic decisions and dictate the necessity of adjuvant chemotherapy in selected cases. Thus, in the last decades, the prognostic significance of necrosis in resectable non-small cell carcinomas has been assessed by many investigators in a series of, mostly retrospective, studies investigating the impact of these histological features on survival of patients. (Lipford et al. 1984; Elson et al. 1988; Lee et al. 1989; Roeslin et al. 1991; Roberts et al. 1992; Shahab et al. 1992; Tormanen et al. 1995; Kessler et al. 1996; Goldstein et al. 1999; Swinson et al. 2002; Poleri et al. 2003; Khan et al. 2004; Rena et al. 2007; Inoue et al. 2008; Cho et al. 2008; Sardari Nia et al. 2010; Kiliçgün et al. 2010; Park et al. 2011; Pataer et al. 2012; Tantraworasin et al. 2013).

In the majority of these previous studies, clinical, pathological and follow up data were correlated to tumor necrosis. In 7 studies (Elson et al. 1988; Shahab et al. 1992; Goldstein et al. 1999; Poleri et al. 2003; Rena et al. 2007; Cho et al. 2008; Tantraworasin et al. 2013), patients of stages II and III were excluded, while patients of clinical stage III were the target group in a single report (Lee et al. 1989) and clinical stage was not included in the statistical evaluation of one paper (Roberts et al. 1992). In the remaining studies (Lipford et al. 1984; Roeslin et al. 1991; Tormanen et al. 1995; Kessler et al. 1996; Swinson et al. 2002; Khan et al. 2004; Inoue et al. 2008; Sardari Nia et al. 2010; Kiliçgün et al. 2010; Park et al. 2011; Pataer et al. 2012) patients of various clinical stages (I, II, III) were included. Standard morphological criteria were used for the diagnosis and subclassification of NSCLC tumors. Adenocarcinomas followed by squamous cell carcinomas were the prevailing histological types.

In most of these studies, the presence and extent of microscopically recognized necrotic areas was semi quantitatively evaluated. There is, however, a great discrepancy in the exact scale used for this measurement. In a minority of studies only the presence or absence of necrosis was recorded and no measurement scale was used (Rena et al. 2007; Inoue et al. 2008). Other researchers arbitrarily used the percentage of necrosis in the examined neoplastic tissue – more than 50% (Shahab et al. 1992), more than one third (Khan et al. 2004) more than 20% (Roeslin et al. 1991) and more than 10% (Kessler et al. 1996) of the tissue – as a criterion for classifying the tumor as necrotic. Histological sections from the periphery of the tumor were examined in one study (Swinson et al. 2002) evaluating the influence of the tumor microvasculature on tumor necrosis. There was no sampling from the rest of the neoplastic mass in order to avoid the bias of assessing necrosis secondary to acute infarction. In eight studies (Lipford et al. 1984; Roeslin et al. 1991; Shahab et al. 1992; Goldstein et al. 1999; Poleri et al. 2003; Khan et al. 2004; Inoue et al. 2008; Sardari Nia et al. 2010), no correlation of tumor necrosis with survival was found at univariate statistical analysis; in contrast, the prognostic significance of necrosis was statistically established in seven other retrospective studies (Elson et al. 1988; Roberts et al. 1992; Tormanen et al. 1995; Cho et al. 2008; Kiliçgün et al. 2010; Park et al. 2011; Pataer et al. 2012).

Kessler et al. (Kessler et al. 1996) previously reported that tumor necrosis was associated with a poor overall survival in univariate statistical analysis, but not in multivariate analysis, where only N stage, blood vessel invasion and the T stage were identified as significant prognostic factors. In another study by Törmänen et al. (Tormanen et al. 1995) it was concluded that patients with squamous cell carcinoma showing >20% necrosis had a shorter survival compared to patients with non-necrotic tumors. This finding was confirmed both in univariate and multivariate statistical analyses, while no such correlation was found in cases of lung adenocarcinomas.

Extensive tumor necrosis was found to be of independent prognostic value in a study of Swinson et al. (Swinson et al. 2002), and the authors concluded that in patients with lung cancer of clinical stage I and II, necrosis was useful in improving the predictive power of the TNM staging system. However, the prognostic value of this histological parameter was diminished in cases of patients with clinical stage III, while no association was found between tumor necrosis and angiogenesis. A dismal prognosis of NSCLC patients with tumor necrosis was demonstrated by Kiliçgün et al. (Kiliçgün et al. 2010), both in in univariate and multivariate analysis, and the authors proposed adjuvant chemotherapy to be included in the treatment protocol of all patients with tumor necrosis, irrespectively of clinical stage. Tumor necrosis was found to be a significant negative predictor of survival in a study by Elson et al (Elson et al. 1988). This was confirmed by Shahab et al (Shahab et al. 1992) in a subsequent morphometric analysis of 28 surgically resected tumors from patients with NSCLC.

Necrosis has been previously correlated with tumor recurrence in seven studies (Lee et al. 1989; Kessler et al. 1996; Swinson et al. 2002; Inoue et al. 2008; Sardari Nia et al. 2010; Park et al. 2011; Tantraworasin et al. 2013) and with metastatic disease in three studies (Lee et al. 1989; Roeslin et al. 1991; Swinson et al. 2002). Inoue et al. (Inoue et al. 2008) reported that microscopic necrosis constituted a significant prognostic factor for tumor recurrence in univariate, but not in multivariate analysis. However, a significant correlation of microscopic necrosis with nodal involvement, which was found to be an independent predictor for postoperative recurrence in multivariate analysis, was also demonstrated in the latter study. In another study by Park et al. (Park et al. 2011), tumor necrosis was found to be a significant adverse risk factor for recurrence, both in univariate and in multivariate analysis, and was also correlated with reduced overall survival in univariate analysis. Finally, Tantraworasin et al. (Tantraworasin et al. 2013) identified tumor necrosis along with intratumoral blood vessel invasion, tumor diameter >5 cm and nodal involvement as independent prognostic factors for tumor recurrence, and further reported a positive correlation between tumor necrosis and tumor size (64% of tumors >5 cm in diameter had necrosis in comparison to 30% of tumors <5 cm).

### Apoptotic index

Previous data on the prognostic significance of apoptotic index in NSCLC are rather limited and mostly controversial. Tanaka et al. (Tanaka et al. 1999) calculated the AI using the TUNEL method in a series of surgically resected NSCLCs. Multivariate analysis showed that a moderate AI value predicted a poor survival, while prognosis was relatively good in the lowest-AI group. Interestingly, the highest-AI group had the most favorable prognosis, a fact that was explained by the hypothesis that intense apoptosis overcomes cell proliferation and retards tumor growth. Similar results were observed by other researchers as well, indicating that high AI may be a marker of favorable prognosis in NSCLC (Matturi et al. 1999; Hwang et al. 2001). Matturri et al (Matturi et al. 1999) found that high AI values were associated with improved survival in patients with NSCLC, while Hwang et al (Hwang et al. 2001) reported that a low level of spontaneous apoptosis in pretreatment histology predicted a poor prognosis for radiation-treated NSCLC patients.

In contrast to these findings, Tormanen et al. (Tormanen et al. 1995) reported that an AI higher than 1.5% constituted an independent prognostic factor of shortened survival. In another study by Langendijk et al (Langendijk et al. 2000), high AI was associated with worse local control, more distant metastases and a significantly worse overall survival in patients with inoperable NSCLC treated with high-dose radiotherapy. Furthermore, Dworakowska et al (Dworakowska et al. 2009) found that NSCLC patients with very high AI values combined with very high PCNA (proliferating cell nuclear antigen) labeling index had a particularly poor prognosis and concluded that joint analysis of several apoptosis, proliferation and cell cycle regulation biomarkers may provide more useful prognostic information as compared to AI alone. On the other hand, other researchers failed to reveal any association between AI and survival in patients with NSCLC (Gorgoulis et al. 2000). In the study by Puglisi et al (Puglisi et al. 2002), AI alone had no significant impact on survival, but the combination of low AI and high proliferation activity (high MIB-1 index) was associated with a worse clinical outcome both in univariate and in multivariate analysis. Interestingly, in another study by Komaki et al (Komaki et al. 1996), although apoptosis did not predict survival or distant metastases among all patients with stage N1 NSCLC, high apoptosis predicted significantly better survival in patients with squamous carcinomas (SQ) and significantly worse survival in patients with adenocarcinomas/large cell carcinomas.(AC/LC). Therefore, the authors of the latter study suggested that pretreatment levels of apoptosis might be useful for predicting treatment outcome and metastatic incidence of AC/LC, respectively, when these different lung cancer subtypes are separately analyzed.

## Conclusions

Although several previous clinicopathological studies have demonstrated that tumor necrosis may be associated with an aggressive tumor behavior and reduced survival of patients, the independent prognostic significance of this biomarker in NSCLC has not been firmly established yet. Furthermore, these previous results should be interpreted with caution and viewed in light of certain limitations, mainly including the small sample size and retrospective nature of most series, the variable distribution of the clinicopathological and treatment characteristics of the studied populations as well as the discrepancy in the criteria used by different research groups to define the presence and extent of microscopically recognized necrotic tissue. Nevertheless, it should be emphasized that in a limited number of studies with a significantly large sample size, necrosis was statistically recognized as an indisputable prognostic factor in multivariate analysis.

With regard to the prognostic significance of apoptotic index in NSCLC, previous clinicopathological studies have yielded contradictory and mixed results. Different research groups have reported that a high apoptotic rate may be associated with either shorter or longer survival, while other studies have failed to reveal any statistically significant correlation between AI and prognosis. As previously suggested, these contradictory reports may reflect the fact that the apoptotic process is also influenced by a variety of other prognostic biomarkers, including apoptosis regulating proteins, suppressor oncogene products and a variety of other markers involved in cell cycle regulation or proliferative activity of tumors (Dobashi et al. 2004). Most importantly, the clinical, histological and molecular heterogeneity of lung carcinomas grouped under the term NSCLC may also explain, at least to a certain degree, the failure of previous studies to obtain consistent and reproducible results on the prognostic significance of AI in this form of cancer.

Carefully designed studies with strict criteria concerning the amount of tumor examined and the exact determination of the percentage of the necrotic tissue must be carried out prospectively in the future in order to eligibly define the precise role of necrosis in the final outcome of patients with resectable NSCLC. In addition, the correlation of tumor necrosis with factors such as tumor size, blood vessel invasion, lymphatic invasion and degree of tumor differentiation needs further investigation. Larger studies are also required to highlight the exact role of apoptosis in the determination of the overall survival of patients with NSCLC. Unique histological methodology, proper selection of patients and high statistical power of prospective studies are expected to contribute to the precise definition of the prognostic value of necrosis and apoptosis.

With the advent of patient-tailored targeted therapies in oncology, the need for more accurate risk stratification of patients and improved prediction and monitoring of response to treatment has become more pronounced (Ulivi & Silvestrini 2013). Development of novel targeted agents for NSCLC, modulating pathways involved in cell proliferation, apoptosis, angiogenesis and invasion, is now underway, holding promise for a significant improvement in the management of this devastating disease (Sun et al. 2007). In this regard, carefully selected biomarkers, including apoptosis-related markers such as the AI, may serve in the future not only for diagnosis or prognosis of NSCLC, but also as tools to determine if a new agent is successfully targeting neoplastic cells or not in each individual patient, thus guiding optimal decision-making for personalized therapeutics (Diaz et al. 2008).

## References

[CR1] Cho S, Sung SW, Jheon S, Chung JH (2008). Risk of Recurrence in Surgically Resected Stage I Adenocarcinoma of the Lung: Histopathologic and Immunohistochemical Analysis. Lung.

[CR2] Diaz D, Prieto A, Reyes E, Barcenilla H, Monserrat J, Alvarez-Mon M (2008). Flow cytometry enumeration of apoptotic cancer cells by apoptotic rate. Methods Mol Biol.

[CR3] Dobashi Y, Goto A, Fukayama M, Abe A, Ooi A (2004). Overexpression of CDK4/Cyclin D1, a possible mediator of apoptosis and an indicator of prognosis in human primary lung carcinoma. Int J Cancer.

[CR4] Dworakowska D, Jassem E, Jassem J, Karmolinski A, Lapinski M, Tomaszewski D, Rzyman W, Jaskiewicz K, Sworczak K, Grossman AB (2009). Prognostic value of the apoptotic index analyzed jointly with selected cell cycle regulators and proliferation markers in non-small cell lung cancer. Lung Cancer.

[CR5] Edinger AL, Thompson CB (2004). Death by design: apoptosis, necrosis, and autophagy. Curr Opin Cell Biol.

[CR6] Elson CE, Roggli VL, Vollmer RT, Greenberg SD, Fraire AE, Spjut HJ, Yesner R (1988). Prognostic indicators for survival in stage I carcinoma of the lung: a histologic study of 47 surgically resected cases. Mod Pathol.

[CR7] Goldstein NS, Mani A, Chmielewski, Welsh R, Pursel S (1999). Prognostic factors in T1 NO MO adenocarcinomas and bronchioloalveolar carcinomas of the lung. Am J Clin Pathol.

[CR8] Gorgoulis VG, Kotsinas A, Zacharatos P, Mariatos G, Liloglou T, Tsoli E, Kokotas S, Fassoulas C, Field JK, Kittas C (2000). Association of allelic imbalance at locus D13S171 (BRCA2) and p53 alterations with tumor kinetics and chromosomal instability (aneuploidy) in nonsmal cell lung carcinoma. Cancer.

[CR9] Hwang JH, Lim SC, Kim YC, Park KO, Ahn SJ, Chung WK (2001). Apoptosis and bcl-2 expression as predictors of survival in radiation-treated non-small-cell lung cancer. Int J Radiat Oncol Biol Physic.

[CR10] Inoue M, Takakuwa T, Minami M, Shiono H, Utsumi T, Kadota Y, Nasu T, Aozasa K, Okumura M (2008). Clinicopathologic factors influencing postoperative prognosis in patients with small-sized adenocarcinoma of the lung. J Thorac Cardiovasc Surg.

[CR11] Kessler R, Gasser B, Massard G, Roeslin N, Meyer P, Wihlm JM, Morand G (1996). Blood vessel invasion is a major prognostic factor in resected non-small cell lung cancer. Ann Thorac Surg.

[CR12] Khan OA, Fitzgerald JJ, Field ML, Soomro I, Beggs FD, Morgan WE, Duffy JP (2004). Histological determinants of survival in completely resected T1-2N1M0 nonsmall cell cancer of the lung. Ann Thorac Surg.

[CR13] Kiliçgün A, Turna A, Sayar A, Solak O, Urer N, Gürses A (2010). Very important histopathological factors in patients with resected non-small cell lung cancer: necrosis and perineural invasion. Thorac Cardiovasc Surg.

[CR14] Komaki R, Fujii T, Perkins P, Ro JY, Allen PK, Mason KA, Mountain CF, Milas L (1996). Apoptosis and mitosis as prognostic factors in pathologically staged N1 nonsmall cell lung cancer. Int J Radiat Oncol Biol Phys.

[CR15] Kumar V, Abbas AK, Fausto N, Mitchell RN (2007). Robbins Basic Pathology.

[CR16] Langendijk H, Thunnissen E, Arends JW, de Jong J, ten Velde G, Lamers R, Guinee D, Holden J, Wouters M (2000). Cell proliferation and apoptosis in stage III inoperable non-small cell lung carcinoma treated by radiotherapy. Radiother Oncol.

[CR17] Lee TK, Horner RD, Silverman JF, Chen YH, Jenny C, Scarantino CW (1989). Morphometric and morphologic evaluations in stage III non-small cell lung cancer. Prognostic significance of quantitative assessment of infiltrating lymphoid cells. Cancer.

[CR18] Lipford EH, Eggleston JC, Lillemoe KD, Sears DL, Moore GW, Baker RR (1984). Prognostic factors in surgically resected limited-stage nonsmall cell carcinoma of the lung. Am J Surg Pathol.

[CR19] Matturi L, Colombo B, Lavezzi AM (1999). Relationship with cell kinetics and apoptosis. Anal Quant Cytol Histol.

[CR20] Murray CJ, Lopez AD (2013). Measuring the global burden of disease. N Engl J Med.

[CR21] Park SY, Lee HS, Jang HJ, Lee GK, Chung KY, Zo JI (2011). Tumor necrosis as a prognostic factor for stage IA non-small cell lung cancer. Ann Thorac Surg.

[CR22] Pataer A, Kalhor N, Correa AM, Raso MG, Erasmus JJ, Kim ES, Behrens C, Lee JJ, Roth JA, Stewart DJ, Vaporciyan AA, Wistuba II, Swisher SG (2012). Histopathologic response criteria predict survival of patients with resected lung cancer after neoadjuvant chemotherapy. J Thorac Oncol.

[CR23] Poleri C, Morero JL, Nieva B, Vázquez MF, Rodríguez C, de Titto E, Rosenberg M (2003). Risk of recurrence in patients with surgically resected stage I non-small cell lung carcinoma: histopathologic and immunohistochemical analysis. Chest.

[CR24] Poon IK, Hulett MD, Parish CR (2010). Molecular mechanisms of late apoptotic/necrotic cell clearance. Cell Death Differ.

[CR25] Proskuryakov SY, Gabai VL (2010). Mechanisms of Tumor Cell Necrosis. Curr Pharm Des.

[CR26] Puglisi F, Minisini AM, Aprile G, Barbone F, Cataldi P, Artico D, Damante G, Beltrami CA, Di Loreto C (2002). Balance between cell division and cell death as predictor of survival in patients with non-small-cell-lung cancer. Oncology.

[CR27] Rena O, Carsana L, Cristina S, Papalia E, Massera F, Errico L, Bozzola C, Casadio C (2007). Lymph node isolated tumor cells and micrometastases in pathological stage I non-small cell lung cancer: prognostic significance. Eur J Cardiothorac Surg.

[CR28] Roberts TE, Hasleton PS, Musgrove C, Swindell R, Lawson RA (1992). Vascular invasion in non-small cell lung carcinoma. J Clin Pathol.

[CR29] Roeslin N, Warter, Gasser B, Chakfe N, Weil G, Dumont P, Wihlm JM, Morand G, Witz JP (1991). Non-aplastic N2 operated bronchial cancers Multifactorial analysis of the prognosis. Ann Chir.

[CR30] Sardari Nia P, Van Marck E, Weyler J, Van Schil P (2010). Prognostic value of a biologic classification of non-small-cell lung cancer into the growth patterns along with other clinical, pathological and immunohistochemical factors. Eur J Cardiothorac Surg.

[CR31] Shahab I, Fraire AE, Greenberg SD, Johnson EH, Langston C, Roggli VL (1992). Morphometric quantitation of tumor necrosis in stage 1 non-small cell carcinoma of lung: prognostic implications. Mod Pathol.

[CR32] Sun S, Schiller JH, Spinola M, Minna JD (2007). New molecularly targeted therapies for lung cancer. J Clin Invest.

[CR33] Swinson DE, Jones JL, Richardson D, Cox G, Edwards JG, O’Byrne KJ (2002). Tumour necrosis is an independent prognostic marker in non-small cell lung cancer: correlation with biological variables. Lung Cancer.

[CR34] Tanaka F, Kawano Y, Li M, Takata T, Miyahara R, Yanagihara K, Ohtake Y, Fukuse T, Wada H (1999). Prognostic Significance of Apoptotic Index in Completely Resected Non–Small-Cell Lung Cancer. J Clin Oncol.

[CR35] Tantraworasin A, Saeteng S, Lertprasertsuke N, Arreyakajohn N, Kasemsarn C, Patumanond J (2013). Prognostic factors of tumor recurrence in completely resected non-small cell lung cancer. Cancer Manag Res.

[CR36] Tormanen U, Eerola AK, Rainio P, Vahakangas K, Soini Y, Sormunen R, Bloigu R, Lehto VP, Paakko P (1995). Enhanced apoptosis predicts shortened survival in non-small cell lung carcinoma. Cancer Research.

[CR37] Ulivi P, Silvestrini R (2013). Role of quantitative and qualitative characteristics of free circulating DNA in the management of patients with non-small cell lung cancer. Cell Oncol.

